# Cbl-b negatively regulates TLR/MyD88-mediated anti-*Toxoplasma gondii* immunity

**DOI:** 10.1128/spectrum.00074-23

**Published:** 2023-11-01

**Authors:** Haixia Wei, Shuizhen Wu, Liying Mai, Lili Yang, Weihao Zou, Hongjuan Peng

**Affiliations:** 1 Department of Pathogen Biology, Guangdong Provincial Key Laboratory of Tropical Medicine, School of Public Health, Southern Medical University, Guangzhou, Guangdong, China; 2 Department of Pathogen Biology, School of Basic Medicine, Guangzhou Medical University, Guangzhou, Guangdong, China; University of Camerino, Camerino, Italy

**Keywords:** *Toxoplasma gondii*, TLRs/MyD88, Cbl-b, ubiquitination

## Abstract

*Toxoplasma gondii* is an important intracellular protozoan, which needs to exploit host nutrition for successful parasitism. We previously reported that Casitas B-lineage lymphoma-b (Cbl-b) was screened as a host dependency factor of *T. gondii* by using lentiviral CRISPR-Cas9-single guide RNA (sgRNA) libraries. Furthermore, the detailed mechanism of Cbl-b being required by *T. gondii* infection was explored in this study. The proliferation of *T. gondii* was found to be significantly inhibited in Cbl-b knockdown cell lines, and the Cbl-b expression level increased with prolonged *T. gondii* infection, while the MyD88 level was significantly decreased in the *T. gondii* infection group. Toll-like receptor (TLR)/MyD88 is a conserved innate cellular immune signaling pathway against pathogens infection. Cbl-b was found to interact with MyD88 and mediate MyD88 ubiquitination in the fluorescence resonance energy transfer and co-immunoprecipitation experiments. A Cbl-b knockout (KO) C57BL/6J lineage was then constructed and, together with the wild-type (WT) mice, was infected with the same amount of *T. gondii* tachyzoites of ME49 strain. At 13 days post infection, all the mice in the WT group died, while 80% of the mice in the Cbl-b KO group still survived, even to the end of the experiment. The parasitic burden in the liver, lung, and brain of the Cbl-b KO mice was significantly lower than that of the WT mice (*P* < 0.05). In Cbl-b KO infected mice, the percentage of B cells was higher, whereas that of macrophages was lower, and the interferon-γ and interleukin-6 levels in the serum were higher than that in the WT infected mice; the difference was significant (*P* < 0.05). Furthermore, when host cells were infected by *T. gondii*, host’s Cbl-b was found to interact with MyD88 to ubiquitinate MyD88 for ribosome-dependent degradation. Therefore, the host’s innate immunity against *T. gondii* through the TLR/MyD88 pathway was negatively regulated by Cbl-b.

**IMPORTANCE:**

This is the first report that a human E3 ubiquitin ligase, Casitas B-lineage lymphoma proto-oncogene B (Cbl-b), functions as a host dependency factor for the intracellular protozoan *Toxoplasma gondii* and the mechanism for how *T. gondii* infection inhibits the TLR/MyD88 innate immunity pathway through MyD88 degradation mediated by Cbl-b. This finding is an impactful contribution for understanding the host cell immunity against *T. gondii* infection.

## INTRODUCTION

The intracellular protozoan *Toxoplasma gondii* is widely distributed, and about 30% of worldwide population is serum positive with this parasite (1). Its infection results in non-specific symptoms in immune-competent individuals and serious diseases such as encephalitis and retinochoroiditis in immunocompromised patients (2). As an obligatory parasite, *T. gondii* has to exploit host factors for successful parasitism. In our previous study, Casitas B-lineage lymphoma proto-oncogene B (Cbl-b) was identified as one of the host dependency factors required by *T. gondii*, by using genome-wide CRISPR/Cas9 screening (3). It is found that in the host cells with Cbl-b knockdown, the survival and reproduction rates of the parasites are decreased, while the cells can still survive under the pressure of *T. gondii* infection (3). Cbl proteins are a small class of E3 ubiquitin ligases, which are important negative regulators of immune responses (4). Cbl-b is a member of Cbl family, which contains a highly conserved N-terminal tyrosine-kinase-binding domain and a RING finger domain, and the C-terminal half contains a ubiquitin-associated domain (5). The ring finger domain mediates the transfer of ubiquitin between E2 ubiquitin-conjugating enzymes and the substrate proteins. The main function of Cbl-b is to ubiquitinate the target proteins, transport the ubiquitinated proteins to the lysosome for degradation, and negatively regulate the related signal pathways (6 – 8). Cbl-b plays a crucial role in immunosuppression by negatively regulating the activation of T cells. The expression of Cbl-b in tumor-infiltrating immune cells is significantly higher than that in normal tissues, and the expression of Cbl-b is significantly lower in the immune cells of the patients with autoimmune diseases such as regulatory T cells from systemic lupus erythematosus (7).

Toll-like receptor (TLR) signaling pathways in host cells play an important role in the immune process against *T. gondii* infection. TLR signaling pathways are mainly divided into MyD88-dependent and MyD88-independent pathways. In fact, all TLR family members are involved in MyD88-dependent signaling pathways except TLR3 (9). It is reported that TLR11/12/2/4 in mouse cells can recognize proteins such as glycosylphosphatidylinositol (GPI) and profilin on the surface of *T. gondii* and then activate MyD88-dependent signal pathway (10 – 12). TLR11 is a pseudogene in human genome (13). Human cells rely on TLR2/4/9/5 to recognize GPI and profilin on the surface of *T. gondii* and then activate MyD88-dependent signal pathway (14). The activated TLR/MyD88 signal pathway can enhance the secretion of interleukin-12 (IL-12), IL-6, IL-8, IL-10, interferon-γ (IFN-γ), tumor necrosis factor α (TNF-α), and so on (15 – 18). It is reported that Cbl-b overexpression in human embryonic kidney cells (HEK293) blocks the interaction of TLR4 and MyD88 to negatively regulate TLR4-MyD88-dependent cell signaling in the presence of lipopolysaccharide (LPS) (19). It is also found that Cbl-b interacts with MyD88 and induces the degradation of MyD88 in murine peritoneal macrophages stimulated with LPS (20). In this study, the role and the mechanism of Cbl-b functioning on host’s anti-*Toxoplasma gondii* infection were investigated.

## RESULTS

### The proliferation of *T. gondii* was significantly inhibited by Cbl-b knockdown

**Fig 1:**
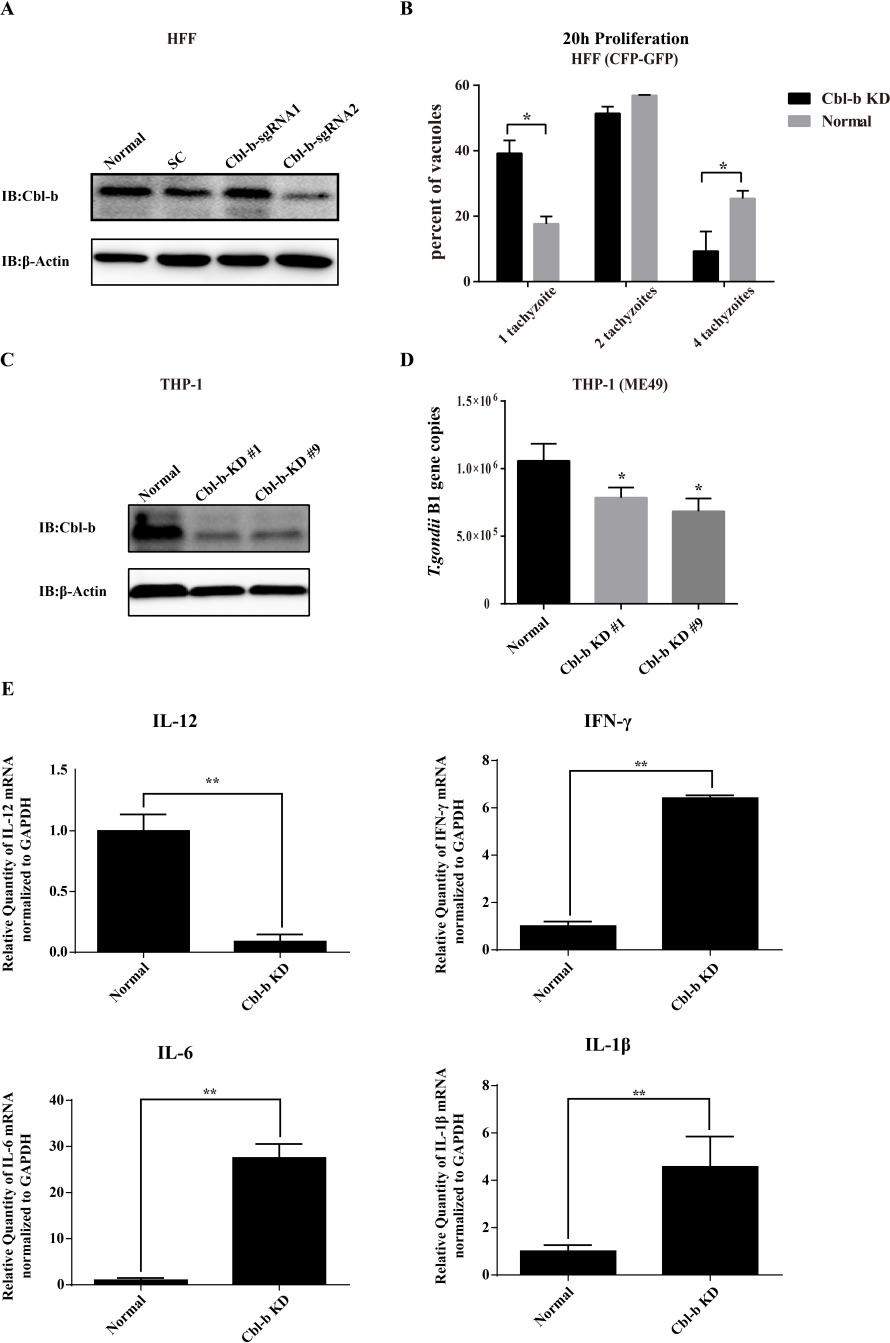
The proliferation of *T. gondii* was significantly inhibited in Cbl-b knockdown cells. (**A**) Western blot detection of Cbl-b in the cell lysates of the HFF cells with different treatments as indicated (normal cells and the cells transfected with SC, Cbl-b-sgRNA1, or Cbl-b-sgRNA2). (**B**) The percentages of the parasitophorous vacuoles containing 1, 2, and 4 tachyzoites in the WT and Cbl-b knockdown (KD) HFF cells infected with CEP-GFP *T. gondii* strain were counted at 20 h post infection. (**C and D**) Normal THP-1 cells and two clones of THP-1 with Cbl-b KD were harvested; Cbl-b in the cell lysates were detected with WB (**C**); Quantitative PCR was used to detect B1 gene copies in these THP-1 cells infected with *T. gondii* ME49 strain (**D**). (**E**) The transcription levels of IL-12, IFN-γ, IL-6, and IL-1β were detected with qRT-PCR in the normal and Cbl-b KD THP-1 cells infected with ME49 for 24 h, then normalized to that of GAPDH, and compared. The experiments were performed at least three times. The differences between groups were analyzed with Prism using a two-tailed Student’s *t*-test or Mann-Whitney U test. Error bars, SEM. *P < 0.05. IB, immunoblotting.

Human foreskin fibroblast (HFF) and human monocytic cell line (THP-1) cells were edited by the CRISPR Cas 9 genome editing technique. It was found that, in HFF cells, Cbl-b expression was significantly down-regulated in Cbl-b-sgRNA2 (the single guide RNA targeting Cbl-b)-transfected group compared to that of the normal cell, Cbl-b-sgRNA1-transfected group and the scrambled sequence-transfected control (SC) group (Fig. 1A). Cbl-b-sgRNA2 was then selected for subsequent experiments.

The normal and Cbl-b knockdown (KD) HFF cells were infected with *T. gondii* CEP-GFP fluorescent strain at multiplicity of infection (MOI) 3. At 20 h post infection (hpi), the percentage of the parasitophorous vacuoles (PVs) containing one parasite in the control group was much higher than that in the Cbl-b KD group (*P* < 0.05), while the percentage of the PVs with four parasites in the control group was much lower than that in the Cbl-b KD group (*P* < 0.05), and no significant difference was found for the percentage of the PVs with two parasites between these two groups (Fig. 1B).

The consistent results were seen in the THP-1 cells. The Cbl-b expression in the Cbl-b KD groups (#1 and #9 clones) was decreased significantly compared to that in the normal cells (Fig. 1C). The normal and Cbl-b KD THP-1 cells were infected with ME49 strain at MOI 1. At 24 hpi, *T. gondii* B1 gene was amplified by quantitative PCR (qPCR) to evaluate the parasitic proliferation. The results showed that the proliferation efficiency of *T. gondii* in Cbl-b KD group was significantly lower than that in the normal group (*P* < 0.05) (Fig. 1D). Furthermore, the transcription of IL-12, IFN-γ, IL-6, and IL-1β in the normal and the Cbl-b KD THP-1 cells upon *T. gondii* infection (MOI = 1) were detected by qRT-PCR. The results showed that the transcription levels of IL-12 in the Cbl-b KD THP-1 cells were lower than that in the normal cells, while the transcription levels of IFN-γ, IL-6, and IL-1β in Cbl-b KD THP-1 cells were higher than that in the normal cells (*P* < 0.01) (Fig. 1E).

### *T. gondii* infection led to enhanced Cbl-b expression and MyD88 degradation

**Fig 2:**
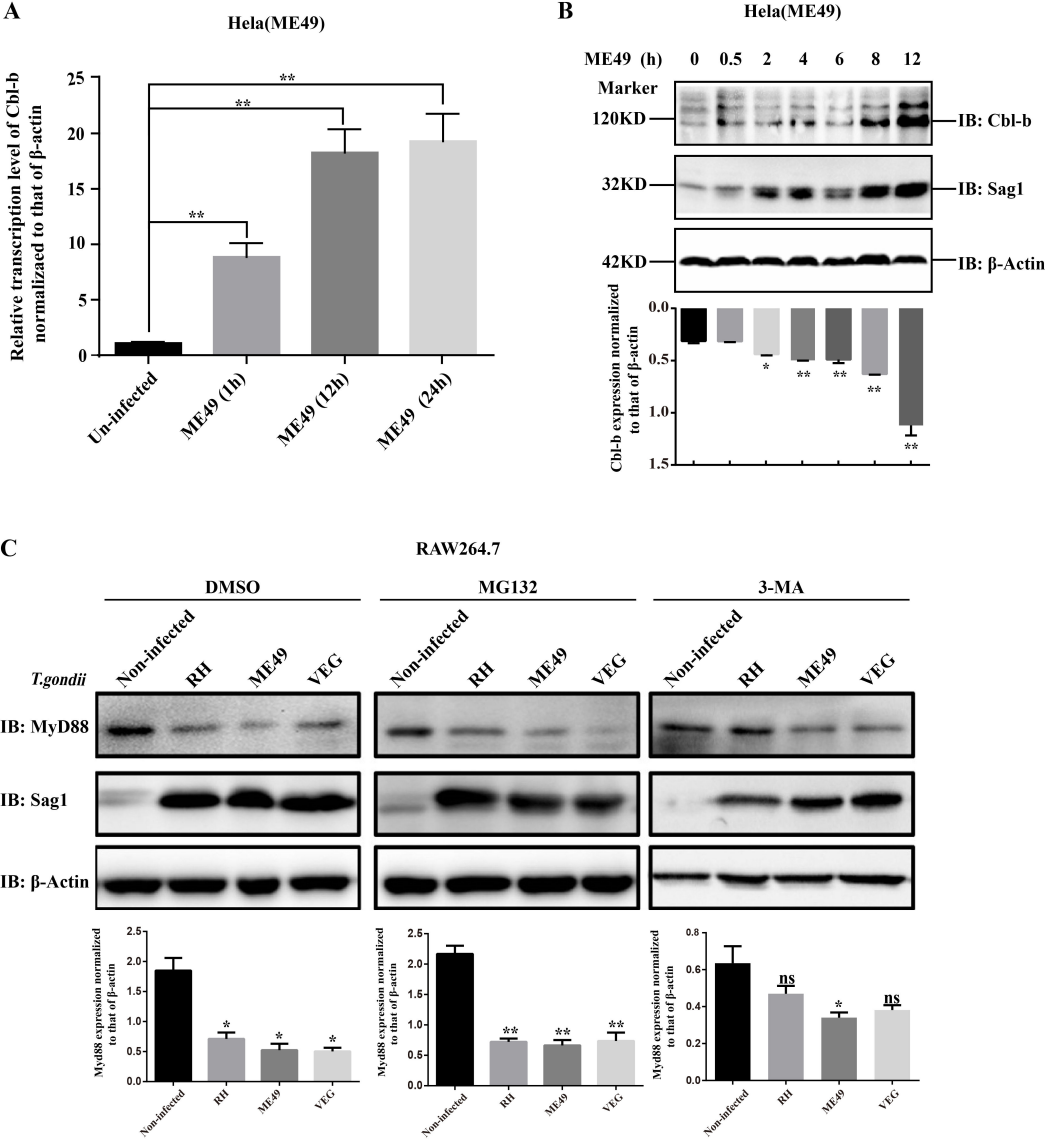
*T. gondii* infection led to enhanced Cbl-b expression and MyD88 degradation. HeLa cells were infected with the same amount of *T. gondii* tachyzoites (MOI = 1). (A) The transcription of Cbl-b in the ME49-infected group at 0 h, 1 h, 12 h, and 24 h post infection was detected with qRT-PCR. (B) WB detection and densitometric analysis for Cbl-b expression at 0.5 h, 2 h, 4 h, 6 h, 8 h, and 12 h post ME49 infection. Sag1 was detected to show the *T. gondii* tachyzoites. (C) Three groups of RAW 264.7 cells were infected with RH, ME49, and VEG strain at MOI 5 for 28 h, respectively, and the uninfected group was used as a control. The four cell groups were set at triplicate and treated with DMSO, MG132, and 3-MA, respectively. The total proteins were harvested and subjected to WB. Then, the expression of MyD88 was detected by WB and densitometric analysis. β-Actin was detected as the loading control in B and C. These experiments were repeated at least three times. The differences between groups were analyzed with Prism using a two-tailed Student’s *t*-test. Error bars, SEM. *P < 0.05.

HeLa cells were infected with ME49 tachyzoites (MOI = 1). The Cbl-b transcription level in the ME49 infection group was increased gradually within the 24 hpi (*P* < 0.01) (Fig. 2A), and the Western blot (WB) showed similar results (Fig. 2B). At 0.5 h, 2 h, 4 h, 6 h, 8 h, and 12 h post infection, the amount of Cbl-b in the infected cells was increased gradually and was larger than that in the uninfected group (*P* < 0.05). The expression level of β-actin was consistent among these seven groups (Fig. 2B). Toll-like receptor (TLR)/MyD88 signaling pathway of host cells plays a key role in the immune process against *T. gondii* infection. RAW264.7 cells were infected with RH, ME49, and VEG tachyzoites at MOI of 5. At 28 hpi, the total protein was harvested and subjected to WB. The results showed that the amount of MyD88 was decreased after *T. gondii* infection (Fig. 2C), and this phenomenon was not changed after adding the proteasome inhibitor MG132. However, the degradation of MyD88 was partially alleviated after adding the lysosomal degradation inhibitor 3-MA (Fig. 2C). These results suggested that MyD88 was degraded through the lysosomal pathway after *T. gondii* infection.

### Cbl-b interacted with MyD88 and mediated the ubiquitination and degradation of MyD88

**Fig 3:**
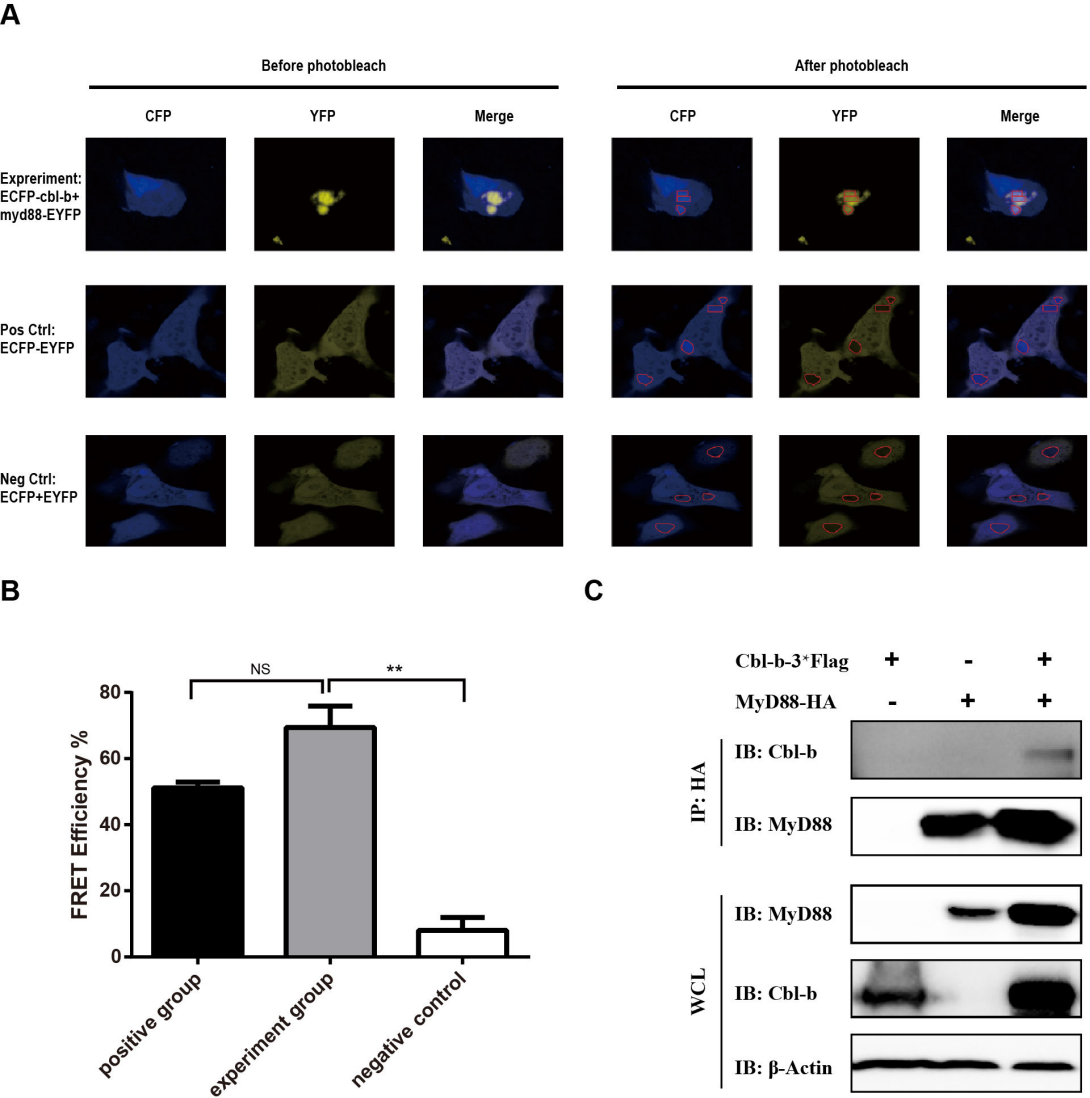
Identification of Cbl-b-MyD88 interaction. (**A and B**) The interaction of Cbl-b and MyD88 was identified with FRET. COS7 cells were transfected with pEYFP-CFP for positive control, co-transfected with pEYFPC1-MyD88 and pECFPN1-Cbl-b for the experimental group, and co-transfected with pEYFP and pECFP for the negative control. The FRET efficiency was analyzed by acceptor photo-bleach technique. Before photo-bleach, the donor CFP fluorescence (F408) and acceptor YFP fluorescence (F514) were tracked upon donor excitation. After photo-bleach, acceptor YFP *in situ* (the red line circled areas) was eliminated without affecting CFP. The extent of dequenching of CFP fluorescence (F408) upon elimination of the energy transfer to YFP is proportional to the FRET efficiency. Ten fields were evaluated. The experiment was performed in triplicate, and the experiments were repeated three times. (**A**) One representative field for each group. (**B**) The FRET efficiencies were compared among the positive control group, the experimental group, and the negative control group. The differences between groups were analyzed with Mann-Whitney U test. Error bars, SEM. **P < 0.01. pcDNA3.1(+)-Cbl-b-3×FLAG and pcDNA3.1(+)-MyD88-HA plasmids were transfected separately or together to COS7 cells on the T75 culture flasks as indicated. The cell lysates were subjected to Co-IP assay, and the anti-HA antibody was used as the bait to capture the proteins interacting with MyD88. β-Actin was detected as the loading control. The experiment was performed at least three times. WCL, whole cell lysate.

The interaction of Cbl-b and MyD88 was detected with fluorescence resonance energy transfer (FRET). The FRET efficiencies were measured by acceptor photo-bleach technique (Fig. 3A). The FRET efficiencies in the positive control group (cells transfected with pEYFP-CFP) and the negative control group (cells co-transfected with pEYFP and pECFP) were about 50% and 10%, respectively, while the FRET efficiency in the experimental group (cells co-transfected with pEYFPC1-MyD88 and pECFPN1-Cbl-b) was about 70%, which was significantly higher than that in the negative control group (*P* < 0.01) (Fig. 3B). pcDNA3.1(+)-Cbl-b-3×FLAG and pcDNA3.1(+)-MyD88-HA plasmids were transfected to COS7 cells separately or together. The co-immunoprecipitation (Co-IP) assay was further used to confirm the interaction of Cbl-b and MyD88. The WB result showed when MyD88 was used as a bait protein, Cbl-b-Flag could be detected in the immunoprecipitants using an anti-Flag antibody only in the dually transfected cells (Fig. 3C), which indicated the interaction of Cbl-b and MyD88.

**Fig 4:**
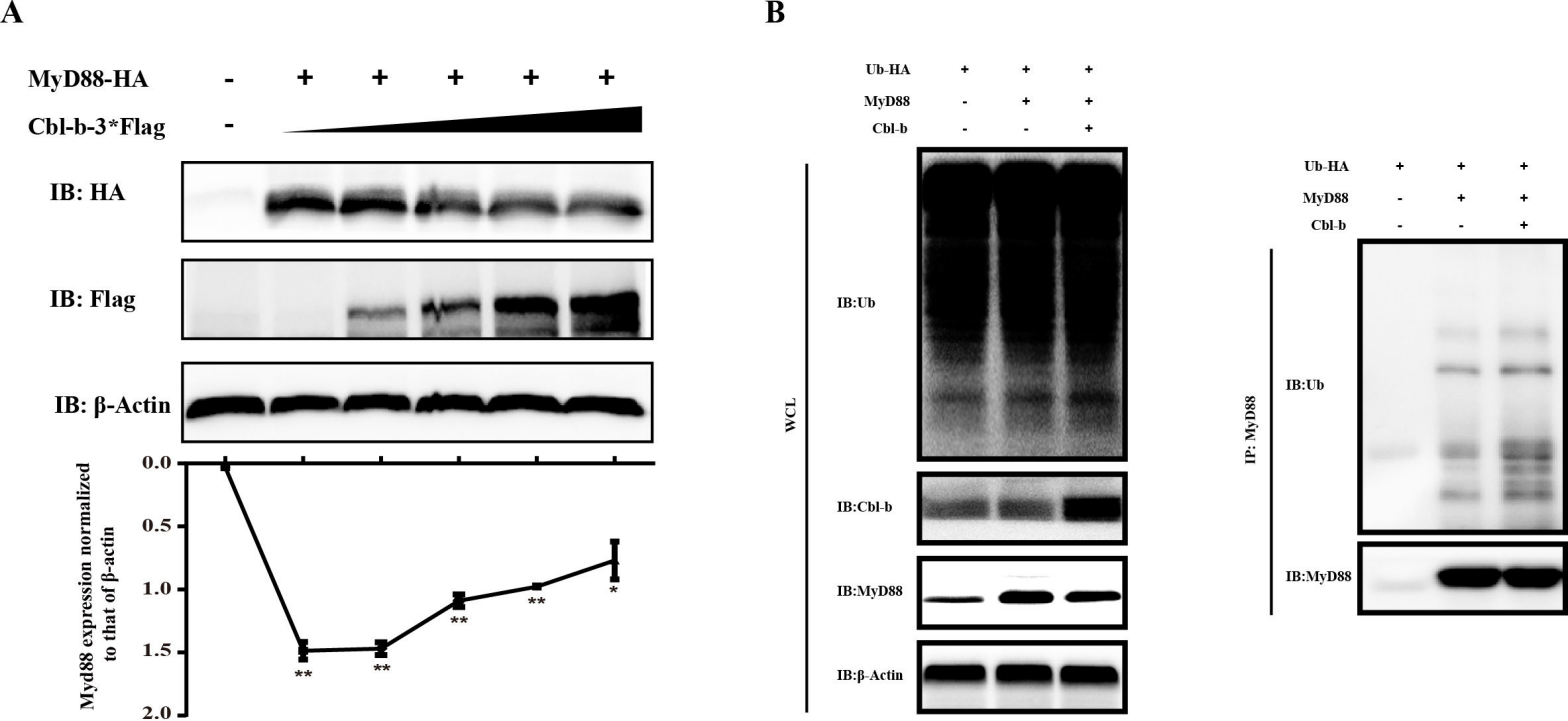
Cbl-b mediated the ubiquitination and degradation of MyD88. (**A**) The same amount (2.0 µg) of pcDNA3.1(+)-MyD88-HA plasmid was co-transfected with 0 µg, 0.5 µg, 1.0 µg, 1.5 µg, and 2.0 µg of pcDNA3.1(+)-Cbl-b-3×FLAG plasmid into COS7 cells in the six-well plate, respectively. The WB result and the densitometric analysis of MyD88 intensity showed that MyD88 protein level decreased with the increased Cbl-b amount. (**B**) Two-microgram pcDNA3.1(+)-Cbl-b-3×FLAG and 2 µg pcDNA3.1(+)-MyD88-HA plasmids were co-transfected to HEK293T cells in T25 culture flasks separately or together as indicated, and 2 µg pcDNA3.1(+)-Ub-HA was transfected into all the groups. After that, the cells were treated with 10 µM MG132 for 12 h. Anti-MyD88 antibody was used as the bait to pull down MyD88, and FK2 antibody was used to detect the ubiquitinated MyD88. β-Actin was detected as the loading control in A and B. These experiments were performed at least three times. The differences between groups were analyzed with Prism using a two-tailed Student’s *t*-test. Error bars, SEM. **P < 0.01.

Furthermore, we found that Cbl-b could mediate the degradation of MyD88. After 2-µg pcDNA3.1(+)-MyD88-HA plasmid was co-transfected with 0 µg, 0.5 µg, 1.0 µg, 1.5 µg, and 2.0 µg of pcDNA3.1(+)-Cbl-b-3×FLAG, respectively, at 48 h post transfection, the WB detection of the cell lysates showed that MyD88 protein level decreased with the increase of Cbl-b amount (Fig. 4A). To further explore the possible reason for MyD88 degradation, the ubiquitinated MyD88 was detected by WB. pcDNA3.1(+)-Cbl-b-3×FLAG and pcDNA3.1(+)-MyD88-HA plasmids were transfected into HEK293T cells separately or together, and pcDNA3.1(+)-Ub-HA was transfected into all the cell groups. Anti-MyD88 antibody was used as the bait to pull down MyD88, and anti-ubiquitinylated protein mouse monoclonal antibody clone FK2 was used to detect the ubiquitinated MyD88. As shown in Fig. 4B, more ubiquitinated MyD88 was found in the co-transfection group than that in the other groups.

### Cbl-b deficiency enhanced host immune response against *T. gondii*

**Fig 5:**
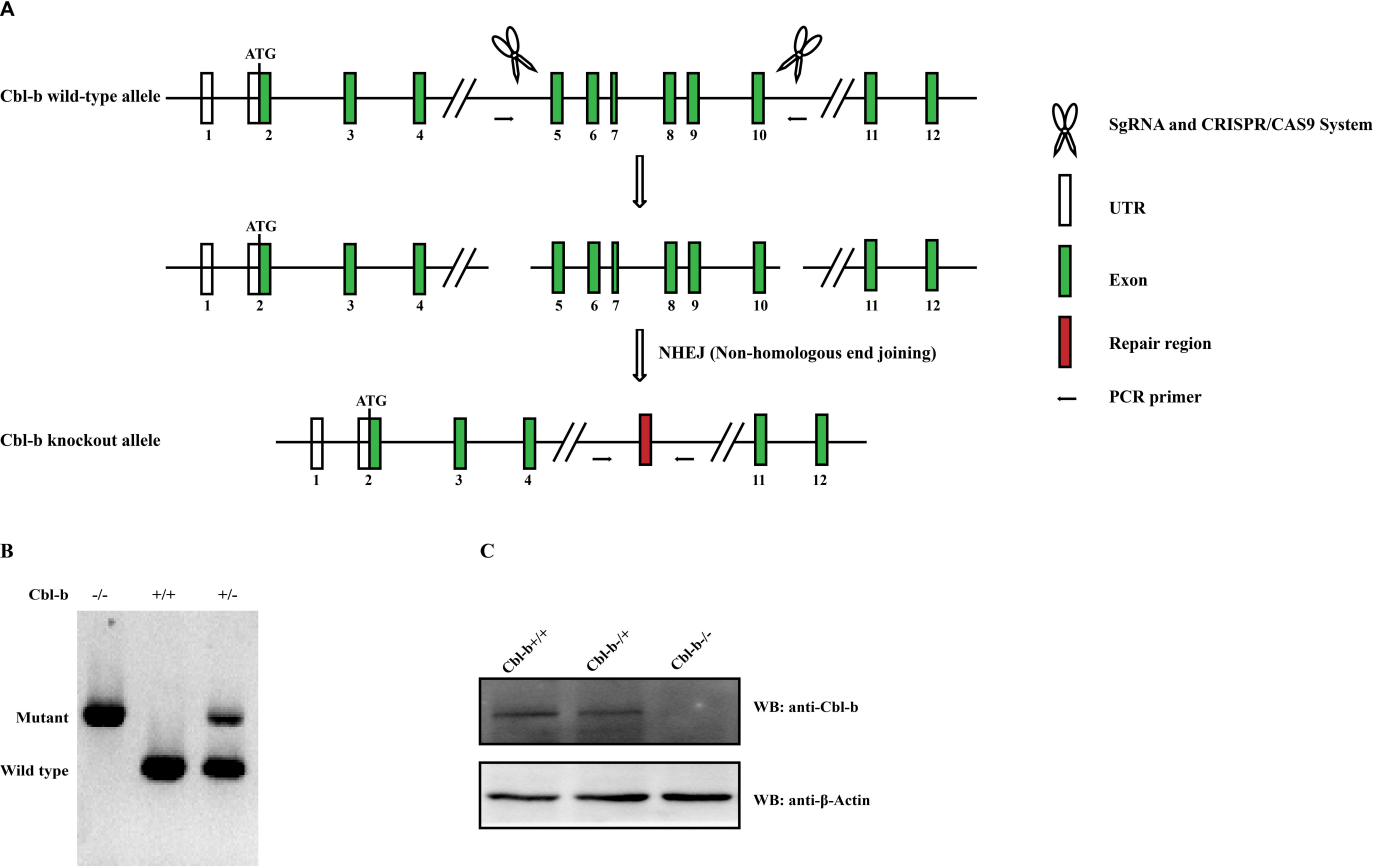
The construction of Cbl-b knockout mice. (**A**) The schematic chart for constructing Cbl-b KO mice. Cbl-b gene has 18 exons, with the ATG start codon in exon 2 and TAG stop codon in exon 18. Two sgRNAs direct Cas9 endonuclease cleavages in intron 4–5 and intron 10–11 of Cbl-b gene and create a double-strand break. Such a break is repaired by non-homologous end joining and results in the disruption of Cbl-b. (**B**) PCR was used to identify the KO of Cbl-b with genome DNA as a template. (**C**) WB was used to identify the KO efficiency of Cbl-b.

To further explore the role of Cbl-b in host’s immunity against *T. gondii*, Cbl-b KO mice on C57BL/6J background were constructed. As shown in Fig. 5A, the Cbl-b gene has 18 exons, with the ATG start codon in exon 2. sgRNAs directed Cas9 endonuclease to cleave Cbl-b gene in intron 4–5 and intron 10–11 and created a double-strand break. Such breaks would be repaired by non-homologous end joining and resulted in the disruption of Cbl-b gene. The pups were genotyped by PCR and WB (Fig. 5B and C).

**Fig 6:**
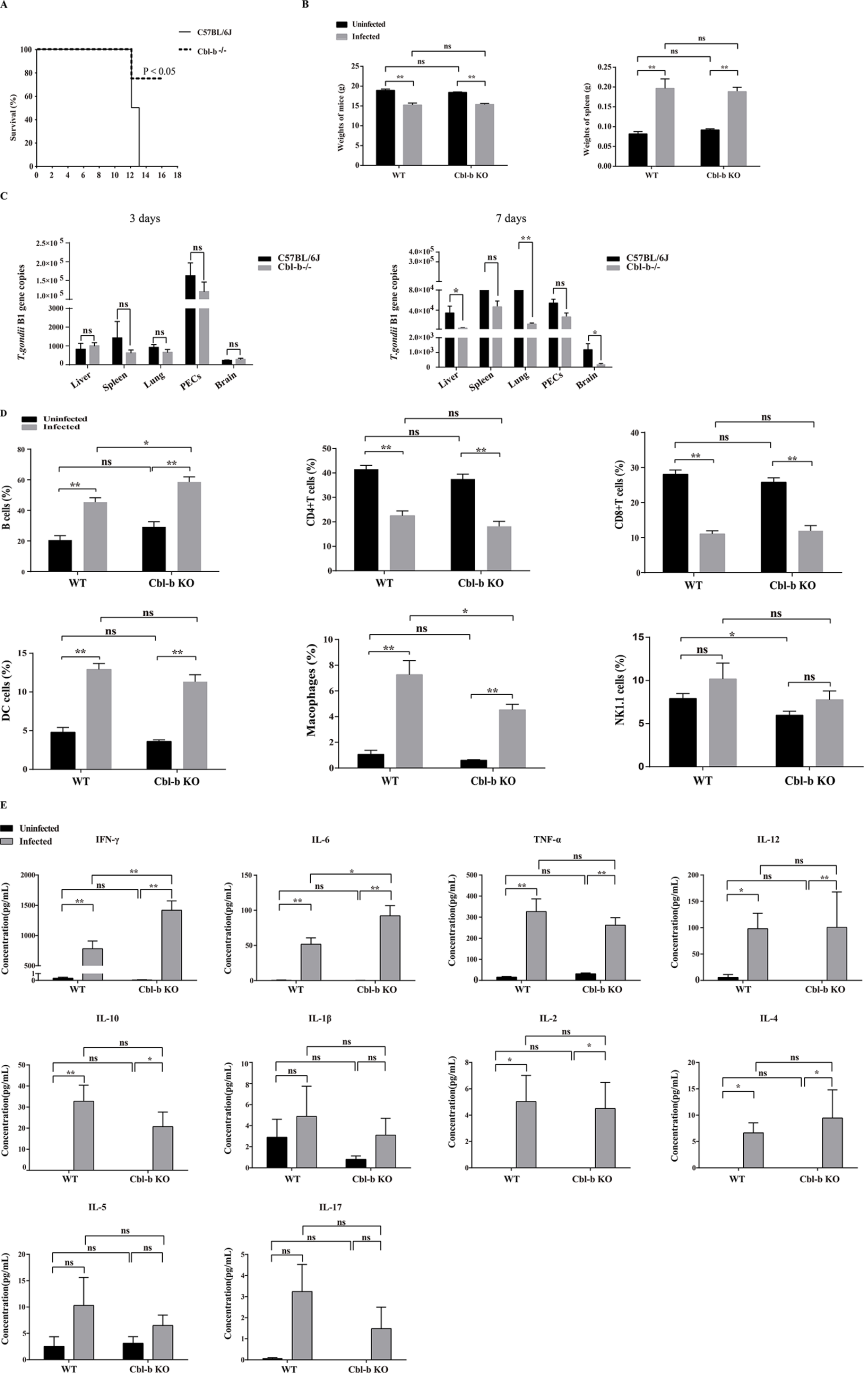
Cbl-b deficiency enhanced host immune response against *Toxoplasma gondii*. Both the C57BL/6J WT and Cbl-b KO mice were intraperitoneally injected with 1,000 *T. gondii* ME49 tachyzoites. (**A**) The survival curves for the C57BL/6J WT and Cbl-b KO mice after infection. (**B**) The infected C57BL/6J WT and Cbl-b KO mice were sacrificed at 7 dpi. Comparison of the weight of the mice and the weight of the spleen between the infected and uninfected groups. (**C**) The infected C57BL/6J WT and Cbl-b KO mice were sacrificed at 3 or 7 dpi. *Toxoplasma gondii* B1 gene’s copy was detected by qPCR to evaluate the parasitic burden in different organs. (**D**) The infected C57BL/6J WT and Cbl-b KO mice were sacrificed at 7 dpi. Comparison of the percentage of the cell subsets as indicated between the infected and uninfected mice after flow cytometry detection. (**E**) The infected C57BL/6J WT and Cbl-b KO mice were sacrificed, and the serum was collected at 7 dpi. The IFN-γ, IL-6, TNF-α, IL-12, IL-10, IL-1β, IL-2, IL-5, IL-17, and IL-4 in the serum of the mice were detected with flow cytometry. Six mice were prepared for each group, and the experiments were repeated at least three times. The differences between groups were analyzed with Prism using a two-tailed Student’s *t*-test or one-way analysis of variance with SPSS software package. Mann-Whitney U test was used with unequal variance or abnormal distributions. Error bars, SEM. *P < 0.05. **P < 0.01. PECs, peritoneal cells.

Both the WT and Cbl-b KO mice were infected with 1,000 *T. gondii* ME49 tachyzoites. At 13 days post infection (dpi), all the WT mice died, while 80% of the Cbl-b KO mice still survived to the end of the experiment (more than 30 dpi) (*P* < 0.05) (Fig. 6A). Significantly decreased weight and enlarged spleen were observed in the *T. gondii*-infected mice, compared to that of the uninfected mice, regardless of whether Cbl-b was knocked out (*P* < 0.01) (Fig. 6B). However, Cbl-b KO made no significant difference on the weight of the mice and the weight of the spleen of the infected and uninfected mice (*P* > 0.05) (Fig. 6B). *T. gondii* B1 gene was amplified by qPCR to evaluate the parasitic burden in different organs. As shown in Fig. 6C, the parasitic burden in different organs was not significantly changed at 3 dpi, while the parasitic burden in the liver, lung, and brain of the Cbl-b KO mice was significantly lower than that in the organs of the WT mice at 7 dpi (*P* < 0.05).

Meanwhile, the immune cell subsets in the *T. gondii*-infected mice were evaluated. As shown in Fig. 6D, in both Cbl-b KO and WT mice, the proportion of B cells, dendritic cells, and macrophages increased in the infected group, while the percentage of CD4^+^ and CD8^+^ T cells decreased in the infected group, compared with that in the uninfected mice (*P* < 0.01). On the other hand, the percentage of B cells was increased, and that of macrophages was decreased in the Cbl-b KO infected mice, compared with that in the WT infected mice (*P* < 0.05). These results indicated that the differentiation and development of B cells and macrophages were regulated by Cbl-b during *T. gondii* infection.

Cytokines play important roles in host’s immune responses against *T. gondii*. The IFN-γ, IL-6, TNF-α, IL-12, IL-10, IL-1β, IL-2, IL-5, IL-17, and IL-4 in the serum of the mice were detected. As shown in Fig. 6E, the level of serum IFN-γ, IL-6, TNF-α, IL-12, IL-10, IL-2, and IL-4 increased in the infected group compared with that in the uninfected group, in both Cbl-b KO and WT mice (*P* < 0.05). Moreover, the levels of IFN-γ and IL-6 were significantly increased in the Cbl-b KO infected mice, compared with that in the WT infected mice (*P* < 0.05) (Fig. 6E). These results indicated that Cbl-b was important for IFN-γ and IL-6 secretion upon *T. gondii* infection.

## DISCUSSION

*T. gondii* is an intracellular parasite with numerous and widespread hosts. In this study, both the transcription and expression level of host Cbl-b were found increased with the time of *T. gondii* infection (Fig. 2A and B). It has been reported that the Cbl-b of peripheral blood mononuclear cells is highly expressed in the patients with helminth-induced chronic infection (21).

It is reported that C-type lectin receptors (CLRs, dectin-1, and dectin-2) play an important anti-infection role in human’s body, but the expression level of this protein is gradually decreased with the extension of the infection time after fungal infection (22 – 25). Cbl-b was thought to be a therapeutic target for fungal infectious diseases. The reason is that when cells are infected by fungi, Cbl-b interacts with and ubiquitinates dectin-1 and dectin-2, leading to their degradation through the lysosomal pathway, to negatively regulate the natural immune response mediated by CLRs (22 – 25). In our previous study, Cbl-b was identified as a host dependence factor with the genome-wide CRISPR-Cas9 library screening technology. When Cbl-b was knocked down or knocked out, the proliferation of *T. gondii* was significantly inhibited (Fig. 1 and 6C). Cbl-b was thought to ubiquitinate and degrade dectin-1 and dectin-2 through lysosomal pathway to negatively regulate the natural immune response mediated by CLRs in *T. gondii* infection (26). However, because of the specificity of *T. gondii* surface antigen, dectin-1 KO did not change the efficiency of *T. gondii* invasion, indicating that CLR signaling pathway did not participate in the host’s immune process against *T. gondii* (26). TLR signaling pathways in host cells play an important role in the immune process against *T. gondii* infection. The activated TLR/MyD88 signal pathway enhances the secretion of IL-12, IL-6, IFN-γ, IL-8, IL-10, TNF-α, and so on (15 – 18). Our study found that Cbl-b interacted with MyD88 and mediated the ubiquitination and degradation of MyD88 (Fig. 2–4). It is reported that Cbl-b interacts with MyD88 and induces the degradation of MyD88 when stimulated by LPS in mouse peritoneal macrophages (20). Altogether, these results suggested that Cbl-b was an important negative regulator for the immune responses mediated through TLR/MyD88 signaling pathway.

Cbl-b KO mice were constructed and used to study the function of Cbl-b. The survival curves of the mice infected by *T. gondii* showed that, compared with the WT mice, Cbl-b KO mice could live longer (Fig. 6A), and the percentage of B cells increased and which of macrophages decreased in Cbl-b KO mice (Fig. 6D). The percentage of B cells and macrophages in the spleen of Cbl-b KO uninfected mice is not significantly different from that in the WT uninfected mice. These results indicated that Cbl-b KO resulted in increase of B cell’s percentage and decrease of macrophage’s percentage after *T. gondii* infection. B cells play a crucial role in humoral immunity which can differentiate into plasma cells, synthesize, and secrete specific antibodies (27). Apart from secreting antibodies, B cells also work as antigen presenting cells for soluble antigens (28). It has been reported that B cells play an important role in host anti-*T*. *gondii* infection and are required for vaccination-induced resistance to virulent tachyzoites (29). All of the B cell-deficient mice complemented with naïve B-1 cells died 18 days after *T. gondii* infection, whereas B cell-deficient mice complemented with primed B-1 cells survived about half a year post infection. More Th1- and Th2-type cytokines and nitric oxide production were found in *T. gondii*-infected B cell-deficient mice complemented with primed B-1 cells (30). We found in our study that the level of IFN-γ, IL-6 were significantly elevated in the infected mice regardless of whether Cbl-b was knocked out, but it was higher in the Cbl-b KO mice than that in the WT mice after *T. gondii* infection (Fig. 6E). These results indicated that Cbl-b functioned to suppress IFN-γ and IL-6 secretion upon *T. gondii* infection. IFN-γ is essential in controlling acute *Toxoplasma gondii* infection and preventing the reactivation of latent bradyzoites (31, 32). IFN-γ is indispensable for host defense as it is essential for the expression of IFN-γ-inducible genes, which leads to clearance of intracellular microbes (33). B cells were reported to be able to promote CD4 and CD8 T cells’ IFN-γ production during Th1 inflammatory response to *T. gondii* infection (34). Taken together, the effect of Cbl-b on mice immune response may be associated with B cells.

**Fig 7:**
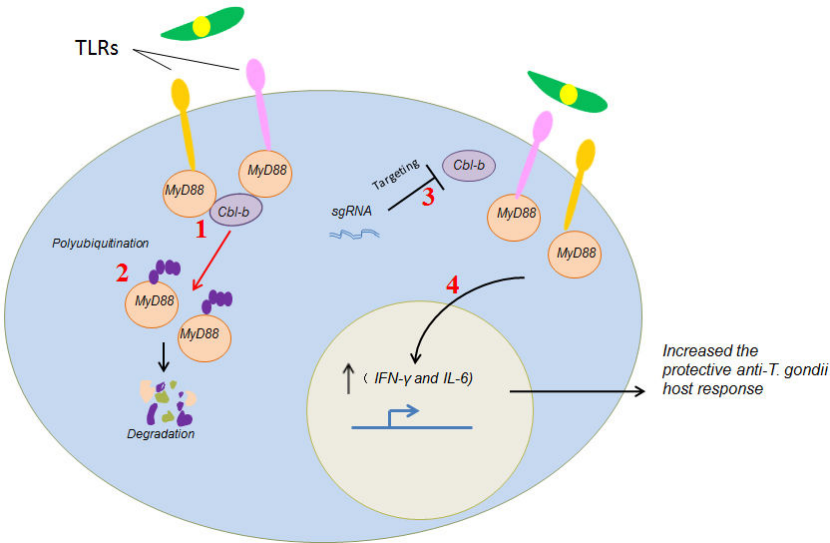
Model for how Cbl-b negatively regulated the anti-*T*. *gondii* innate immunity through TLR/MyD88 signaling. 1, *T. gondii* infection induced the expression of host protein Cbl-b. 2, Furthermore, Cbl-b ubiquitinated MyD88 for degradation, which resulted in the inhibition of anti-infection cytokines (including IFN-γ and IL-6) secretion. By this way, *T. gondii* can survive and multiply successfully in the host cells. 3 and 4, However, when host cell Cbl-b was KO by CRISPR-Cas9 technology, *T. gondii* infection activated TLR/MYD88 signaling pathway to promote the secretion of IL-12, IL-6, IL-8, IL-10, IFN-γ, TNF-α, and so on. As a result, host cell anti*-T*. *gondii* responses are promoted, and tachyzoites can be eliminated.

In summary, our study indicated that Cbl-b negatively regulated the anti-*T*. *gondii* innate immunity mediated by TLR/MyD88 signaling (Fig. 7). *T. gondii* infection induced the expression of host protein Cbl-b, and then Cbl-b ubiquitinated MyD88 for degradation through lysosomal pathway. As a result, the secretion of anti-infection cytokines including IFN-γ and IL-6 was significantly inhibited; *T. gondii*, therefore, can survive and multiply successfully in the host cells.

## MATERIALS AND METHODS

### Parasites and cell lines

The tachyzoites of wild-type *T. gondii* ME49, RH, VEG, and CEP-GFP fluorescent strain were maintained in our laboratory. The HFF, THP-1, COS7, HEK293T, RAW264.7, and HeLa cell lines were purchased from the American Type Culture Collection (USA). The cells were cultured in Dulbecco's Modified Eagle Medium (DMEM medium) (Gibco, New York, NY) containing 10% fetal bovine serum (FBS) (Gibco, New York, NY) in 5% CO_2_ at 37°C. *T. gondii* tachyzoites were cultured in HFF cells in DMEM containing 1% FBS. When most of host cells were ready to be ruptured, the cells were scraped and passed through a syringe several times. The tachyzoites were then purified with a 3-µm filter (Whatman, Kent, UK) (35).

### CRISPR/Cas9-mediated Cbl-b knockdown in human cells

The CRISPR-Cas9 system was used to knock down Cbl-b expression in HFF and THP-1 cells. Human Cbl-b cDNA sequence was obtained from GeneBank, and two sgRNA sequences of Cbl-b -sgRNA1 and Cbl-b -sgRNA2 (shown in Table 1) were designed online (http://www.e-crisp.org/E-CRISP/designcrispr.html). A 20-bp scrambled sequence named “SC” (shown in Table 1) was used as a negative control.

**TABLE 1:** Sequences for the sgRNA

Name of sgRNA	Sequence
Cbl-b-sgRNA1	5′-CGTAAATGCTGATATGTATC-3′
Cbl-b-sgRNA2	5′-ATGAATGGCAGAAACCCTGG-3′
SC	5′-GCACTACCAGAGCTAACTCA-3′

These sequences were ligated into lentiCRISPRv2 by site-directed mutation to create lentiCRISPRv2-Cbl-b-1, lentiCRISPRv2-Cbl-b-2, and lentiCRISPRv2-SC plasmids, respectively. Then, they were transfected into HEK293T cells with lentiviral packaging vectors pCMV-dR8.2 dvpr and pCMV-VSV-G with lipofectamine 2000, respectively. Viruses were collected from the supernatant of the cell culture at 72 h post transfection by passing through a 0.45 filter. HFF and THP-1 cells were infected with the packaged viruses for 72 h in the presence of 10 µg/mL polybrene (Santa Cruz). The cells with stable Cbl-b -sgRNA1, Cbl-b -sgRNA2, or SC expression were selected and maintained with 4 µg/mL puromycin in the further experiments. The expression of Cbl-b in stable cell line was analyzed by WB.

### Detection of *Toxoplasma gondii* proliferation rate

The normal and Cbl-b KD HFF cells were infected with *T. gondii* CEP-GFP fluorescent strain at a multiplicity of infection of 3. At 20 hpi, 100 parasitophorous vacuoles (PVs) were randomly selected under the fluorescence microscope, and the PV numbers containing 1, 2, and 4 tachyzoites were counted respectively, and their composition ratio was used to evaluate the proliferation efficiency.

Similarly, the normal and Cbl-b KD THP-1 cells were infected by *T. gondii* ME49 strain (MOI = 1). At 24 hpi, the infected cells were collected and treated with 0.1 mg/mL proteinase K (Qiagen, Germany). To detect the parasitic burden in different organs of the infected mice, 10-mg spleen and 25-mg other organs or cells were weighted, respectively. Then, the total DNA was extracted. *T. gondii* B1 gene was evaluated by qPCR using the specific primers as previously described (3). The standard curve was obtained through qPCR with B1 gene primers and the plasmid containing B1 gene of known concentrations as the template. Abundance of the parasitic equivalent was determined by extrapolation from the standard curve.

### Real-time PCR

Cell samples were treated with TRIzol (Invitrogen), and then, total RNA was extracted following the manufacturer’s guidance. The cDNAs were synthesized from total RNA with TransScript All-in-One First-Strand cDNA Synthesis SuperMix for qPCR (Transgen Biotech, Beijing, China) using random primers. Quantitative PCR was performed using Hieff qPCR SYBR Green Master Mix (Low Rox Plus) (Yeasen, China) according to the manufacturer’s instruction. Primers for β-actin and target genes were shown in Table S1.

### Western blotting and antibodies

The cells were harvested and lysed. Total protein (20 µg) from the cell lysates of each group was loaded for SDS-PAGE. Proteins in the gel were transferred to polyvinylidene fluoride (PVDF) membrane with 400 mA for 80 min. The PVDF membrane was blocked using 5% bovine serum albumin (BSA) and then incubated in the primary antibodies overnight at 4°C. The membrane was then incubated with secondary antibodies at 37°C for 2 h. Specific proteins on membranes were visualized by Clarity Western ECL Substrate (Bio-Rad, CA, USA).

The following antibodies were used in this study, including anti-Cbl-b mouse polyclonal antibody (Santa Cruz, CA, USA), anti-Sag1 rabbit polyclonal antibody (Abcam, Cambridge, UK), anti-ubiquitinylated protein mouse monoclonal antibody clone FK2 (Millipore Corporation, Billerica, MA, USA), anti-flag M2 mouse monoclonal antibody (Sigma Aldrich, St. Louis, USA), anti-β-Actin rabbit monoclonal antibody (CST, Danvers, MA, USA), anti-MyD88 rabbit monoclonal antibody (CST, Danvers, MA, USA), anti-HA-Tag mouse monoclonal antibody (CST, Danvers, MA, USA), and goat anti-mouse or goat anti-rabbit IgG-HRP (ABclonal Technology, Wuhan, China), fluorescent labeled anti-CD45, CD3, CD19, CD4, CD8, F4/80, NK1.1, CD11c, and CD11b antibodies (BD Biosciences, San Diego, CA, USA).

### Fluorescence resonance energy transfer assay

COS7 cells on coverslips (20 × 20 mm) were co-transfected with 1-µg pECFPN1-Cblb and 1-µg pEYPC1-MyD88 in the experimental group, 1-µg pECFP-N1 and 1-µg pEYFP-C1 as the negative control, and 2-µg pEYFP-CFP as the positive control. At 48 h post transfection, the culture medium was aspirated, and the cells were fixed in 4% paraformaldehyde. The FRET efficiencies from different transfection groups were measured with confocal (FLUOVIEW FV1000; Olympus, Tokyo, Japan).

### Co-immunoprecipitation

COS7 cells grown on the T75 flask were transfected with 5-µg pcDNA3.1(+)-MyD88-HA alone or together with 5μgpcDNA3.1(+)-Cbl-b-3×Flag for 48 h. The cells were washed and lysed. The cell lysate was incubated with anti-HA antibody for 2 h at 4°C. Then, the mix was incubated with protein A-agarose (Santa Cruz Biotechnology, USA) overnight at 4°C with gentle rotation. The agarose beads were collected by centrifugation at 500 × *g* for 5 min. The precipitation samples were analyzed by WB.

### Detection of ubiquitinated MyD88

HEK293T cells grown on the T75 flask were transfected with 2-µg pcDNA3.1-Cbl-b alone or co-transfected with 2-µg pcDNA3.1-MyD88, and 2 µg pcDNA3.1(+)-Ub-HA was transfected into all the groups. Then, the cells were treated with 10 µM MG132 for 12 h. After that, the cells were washed with phosphate buffered saline (PBS) for three times and lysed. The cell lysate was collected and incubated with anti-MyD88 antibody for immunoprecipitation. The samples were subjected to WB with FK2 antibody.

### Generation of Cbl-b KO mice

The Cbl-b KO C57BL/6 mice were constructed by CRISPR/Cas9-mediated genome engineering. The Cblb-201 transcript (ENSMUST00000114471.2) was taken for an example to describe the strategy. Cbl-b gene has 18 exons, with the ATG start codon in exon 2 and TAG stop codon in exon 18. The sgRNAs mediated Cas9 endonuclease cleavage in intron 4–5 and intron 10–11 of Cbl-b gene and created a double-strand break. Such a break will be repaired by non-homologous end joining, and result in the disruption of Cbl-b. The pups will be genotyped by PCR, followed by WB.

### Preparation of splenocytes

Mice were intraperitoneally injected with 1,000 *T. gondii* ME49 tachyzoites and sacrificed at 7 dpi. Spleens were mechanically removed, homogenized, and processed through a 200-µm cell strainer (BD Falcon). Erythrocytes were lysed with RBC lysis buffer (Pythonbio); the cells were washed twice and resuspended in the RPMI-1640 medium containing 10% FBS.

### Splenic lymphocyte analysis with flow cytometry

For cell surface staining, single splenic lymphocytes were washed twice and stained with fluorescein-conjugated anti-CD45, CD3, CD19, CD4, CD8, F4/80, NK1.1, CD11c, and CD11b antibodies for 30 min in the dark. Stained cells were analyzed by flow cytometry (FCM).

### Detection of cytokines in mouse serum with FCM

For detection of cytokines in mouse serum, the samples were assayed using the BD Cytometric Bead Assay (BD Biosciences) Mouse Flex kit according to the manufacturer’s instructions. Serum samples were mixed with the beads to capture the cytokines including IL-2, IL-4, IL-5, IL-6, IL-10, IL-12, IL-17, IL-1β, IFN-γ, and TNF-α, and then, the corresponding antibodies conjugated with phycoerythrin were added. The mix was incubated for 2 h under room temperature in the dark. The beads were collected with centrifugation and washed with PBS for two times and detected by FCM. The mean fluorescence intensity for all cytokines was analyzed.

### Statistics

With equal variance and normal distributions, the differences between groups were analyzed with Prism using a two-tailed Student’s *t*-test or one-way analysis of variance with SPSS software package. Mann-Whitney U test was used with unequal variance or abnormal distributions. The statistical significance was defined as *P* < 0.05.

## SUPPLEMENTAL MATERIAL

Table S1 spectrum.00074-23-s0001.docx Primers used in this research.

ASM does not own the copyrights to Supplemental Material that may be linked to, or accessed through, an article. The authors have granted ASM a non-exclusive, world-wide license to publish the Supplemental Material files. Please contact the corresponding author directly for reuse.
