# Supplementary material for: Cbl-b negatively regulates TLR/MyD88-mediated anti-Toxoplasma gondii immunity
Source: Microbiol Spectr. 2023 Nov 1;11(6):e00074-23. doi: 10.1128/spectrum.00074-23 (PMC10714978; doi:10.1128/spectrum.00074-23)
Supplement: Table S1 — Primers used in this research. [file spectrum.00074-23-s0001.docx]

Table S1 Primers used in this research

| **Name** | **sequences** |
| --- | --- |
| β-actin | 5’-GGACCTGACTGACTACCTCAT-3’  5’-CGTAGCACAGCTTCTCCTTAAT-3’ |
| IFN-γ | 5’-GACCAGAGCATCCAAAAGAGT-3’  5’-ATTGCTTTGCGTTGGACATTC-3’ |
| IL-12 | 5’-GACATTCTGCGTTCAGGTCCAG-3’  5’-CATTTTTGCGGCAGATGACCGTG-3’ |
| IL-6 | 5’-GACTTTAAGGGTTACCTGGGTTG-3’  5’-TCACATGCGCCTTGATGTCTG-3’ |
| IL-1β | 5’-ATGATGGCTTATTACAGTGGCAA-3’  5’-GTCGGAGATTCGTAGCTGGA-3’ |
| Cbl-b | 5’-ACCCTCCTCCCTAGCATAAA-3’  5’-CATCTTCCTCTACTGGGTCTCT-3’ |
